# Prevalence of porcine parvovirus 1 through 7 (PPV1-PPV7) and co-factor association with PCV2 and PRRSV in Korea

**DOI:** 10.1186/s12917-022-03236-1

**Published:** 2022-04-09

**Authors:** Seung-Chai Kim, Jae-Hong Kim, Jae-Yeob Kim, Gyeong-Seo Park, Chang-Gi Jeong, Won-Il Kim

**Affiliations:** College of Veterinary Medicine, Jeonbuk National University, 79 Gobong-ro, Iksan, 54596 Korea

**Keywords:** Pig, Porcine parvoviruses, Porcine circovirus type 2, Porcine reproductive and respiratory syndrome virus, Coinfections, Porcine respiratory disease complex

## Abstract

**Background:**

Classical porcine parvovirus (PPV1) and novel porcine parvoviruses designated porcine parvovirus 2 through 7 (PPV2-PPV7) are widespread in pig populations. The objective of this study was to investigate the prevalence rates of PPV1-PPV7 in Korea by detecting PPVs in serum, lung and fecal samples and to elucidate the association of PPVs with porcine circovirus type 2 (PCV2) and porcine reproductive and respiratory virus (PRRSV), major pathogens involved in porcine respiratory disease complex (PRDC). A total of 286 serum, 481 lung, and 281 fecal samples collected from 2018 to 2020 were analyzed.

**Results:**

The results showed that PPVs are widespread in Korea; the highest detection rates were found in lung samples and ranged from 7.9% (PPV1) to 32.6% (PPV2). Regarding age groups, fattening pigs had the highest detection rates of PPVs, ranging from 6.4% (PPV1) to 36.5% (PPV6); this finding suggests the chronic nature of PPV infections and the continual circulation of these viruses. When compared with PCV2- and PRRSV-negative lung samples, PCV2-positive samples with or without PRRSV positivity had significantly higher detection levels of PPV1 and PPV6. In contrast, the prevalence of PPV2 and PPV7 was significantly higher in PRRSV-infected lung samples regardless of PCV2 detection. PPV5 was detected significantly more frequently in samples  with both PCV2 and PRRSV positivity.

**Conclusions:**

This study could offer a better understanding of the role of PPVs in PCV2 and/or PRRSV infection though further studies are needed to experimentally assess the impact of PPVs in coinfections.

**Supplementary Information:**

The online version contains supplementary material available at 10.1186/s12917-022-03236-1.

## Background

Parvoviruses belonging to the family *Parvoviridae*, which includes the three subfamilies, namely, *Parvovirinae*, which infect vertebrates; *Densovirinae*, which infect arthropods; and *Hamaparvovirinae*, which infects both invertebrate and vertebrate viruses, are small, nonenveloped, single-stranded DNA viruses with a genome of 4–6.3 kb in size [1, 2]. Until recently, porcine parvovirus 1 (PPV1) was solely representative of members of *Parvovirinae* that infect pigs, and PPV1 is one of the most important agents of reproductive failure in the global swine industry [3]. Novel porcine parvoviruses 2 through 7 (PPV2-PPV7) have been described in the last two decades with advances in sequencing technologies such as next-generation sequencing [4–9]. Different studies have revealed that novel PPVs show a wide geographical distribution, and they have been detected in various types of swine samples, including serum, feces, liver, lungs, heart, spleen, kidney, lymph nodes, tonsils and aborted fetuses [4, 6–8, 10–17].

Although PPVs may have a common ancestor, the high genomic variability in PPVs influences their pathogenic potential [18]. Unlike PPV1, the pathogenic role of novel PPVs (PPV2-PPV7) are not yet clearly defined as no experimental challenge has been conducted, and Koch’s postulates remained unfulfilled. Several reports have attempted to identify their importance in pig health. PPV4, PPV6 and PPV7 have been detected in samples from aborted fetuses and adult female pigs and are suspected to cause reproductive failure [8, 11–13]. A high frequency of PPV2 has been observed in lung samples, especially around the onset of respiratory signs [4, 19, 20].

Porcine circovirus type 2 (PCV2) is a prevalent global pathogen that causes numerous types of syndromes and diseases in pigs under the umbrella of PCV2-associated diseases (PCVADs) [21]. In experimental infections with PPV1 and PCV2, the severity of PCVADs and pathological lesions in lymphoid tissues have been shown to increase [22]. Likewise, several studies have been performed to determine the impact of PPV2-PPV7 on PCVADs by focusing on concurrent infections between PPVs and PCV2 from various types of samples and clinical backgrounds [14, 20, 23–28]. However, as different studies have presented different conclusions regarding the association of PPVs with PCV2, the adverse effects that novel PPVs may have on the course of PCV2 infection remain unclear [24].

Regarding PCVAD, PCV2 is suggested to play an important role in the porcine respiratory disease complex (PRDC), which is associated with multiple respiratory pathogens, including porcine reproductive and respiratory virus (PRRSV), swine influenza virus (SIV), and *Mycoplasma hyopneumoniae* [21]. Single or coinfections with PCV2 or/and PRRSV have been found to be the most frequent patterns in PRDC-affected lungs [29]. Novel PPVs have also been detected in PRDC-affected lungs by metagenomics analysis in various studies [29, 30].

All types of PPVs (PPV1-PPV7) have been identified in Korea, but a nationwide investigation of the prevalence of PPVs has not yet been performed [31]. Additionally, PCV2 and PRRSV are known to be highly prevalent not only among Korean swine herds [32, 33], but also among pigs globally [21, 29]. Despite extensive nationwide PCV2 and PRRSV vaccination, PCVAD and PRDC are one of the major clinical threats which result in high economic loss in Korea [32, 34, 35]. As coinfections of PPVs, PCV2 and PRRSV in swine herds are highly suspected, the objective of this study was to investigate the prevalence rates of PPVs together with PCV2 and PRRSV in Korea and to identify the potential relationships of novel PPVs with pathogens (PCV2 and PRRSV) that are related to PRDC in the case of coinfection.

## Methods

### Samples

All the samples utilized were arbitrarily selected and originated from pig case submissions to the Jeonbuk National University Veterinary Diagnostic Center (JBNU-VDC) for diagnostic procedures. A total of 1192 serum samples were collected in 2018 from 61 farms, and the sample size from each farm ranged from 2 to 61 with a mean sample size of 18 samples (25% quartile 10, 75% quartile 25). All the serum samples from the same farm were pooled by 2–5 samples according to their age group prior to nucleic acid extraction. As a result, a total of 268 pooled serum samples were utilized in this study (Table 1). The age groups that were sampled and pooled included piglets under 4 weeks of age (*n* = 39), weaners between 5 and 8 weeks of age (*n* = 91), fatteners from 9 to 25 weeks of age (*n* = 65), and sows or gilts (*n* = 73).

**Table 1:** Information on the number of samples (and farms) used in this study

Collection year	Pooled serum samples	Lung samples	Fecal samples
2018	268 (61)	18 (10)	-
2019	-	195 (92)	251 (79)
2020	-	268 (151)	-
Total	268 (61)	481 (212)	251 (79)

For tissue samples, a total of 481 lung homogenates from 212 farms collected from 2018 to 2020 were included (Table 1). The pigs included piglets (*n* = 28), weaners (*n* = 338), fatteners (*n* = 108), and sows of gilts (*n* = 7). In addition, a total of 251 fecal samples from 79 farms collected in 2019 were utilized (Table 1). The age groups associated with the fecal samples included piglets (n = 45), weaners (*n* = 87), fatteners (*n* = 76), and sows or gilts (*n* = 43).

### Nucleic acid extraction

Lung samples of approximately 0.1 g were minced and diluted 1:10 in phosphate-buffered saline (PBS; 0.1 M, pH 7.4) and homogenized mechanically using the sterile Beadbeater TissueLyser II (Qiagen, Hilden, Germany). Fecal samples of approximately 1 g were processed under the same protocol using a TissueRuptor (Qiagen). The homogenized samples were centrifuged at 2500 rpm for 10 min to obtain supernatant. Total nucleic acids were immediately extracted from the supernatant and serum samples using a PathoGene-spin DNA/RNA Extraction Kit (iNtRON Biotechnology, Inc. Seoul, Korea) according to the manufacturer’s instruction. All the extracts were stored at -80 °C until use.

### PPV detection, capsid protein gene sequencing and phylogenetic analysis

To detect PPVs, a conventional multiplex PCR protocol with published primer sets for detecting all PPV types (PPV1-PPV7) at the same time was utilized to test all the samples as previously described [31]. From PPV-positive samples, 3 or 4 samples were randomly selected from each PPV type, and the capsid protein gene was amplified with newly designed primer sets (Table 2) using the Platinum SuperFi II PCR Master Mix (Invitrogen, Thermo Fisher Scientific, Waltham, MA, USA) according to the manufacturer’s instructions. All the amplicons were purified with the Wizard® SV Gel and PCR Clean-Up Kit (Promega Co., Madison, WI, USA), cloned into the pCR-BLUNT II-TOPO vector using the Zero Blunt TOPO PCR Cloning Kit (Invitrogen), and transformed into XL1-Blue supercompetent cells (Stratagene, San Diego, CA, USA) according to the manufacturer’s instructions. Monoclonal bacterial strains for each cloned product were cultured, and the extracted plasmids (extracted using an Exprep™ Plasmid SV Mini Kit, GeneAll Biotechnology Co., Seoul, Korea) were sequenced using a commercial sequencing service (Macrogen Inc., Seoul, Korea) with additional sequencing primers (Table 2). The capsid protein gene sequences were successfully assembled using Seqman™ (DNASTAR Inc., Madison, WI, USA) and submitted to NCBI GenBank under accession numbers MZ491178 – MZ491200.

**Table 2:** Primers designed for PPV capsid sequencing in this study

Primer	Nucleotide sequence (5'—3')
PPV1_Cap_F	ATAAGGTAGGATGGCGCCTC
PPV1_Cap_R	ACATGATTAACCAAGTAACTGAG
PPV1_Cap_seq_1	GGGAGGGCTTGGTTAGAATCAC
PPV1_Cap_seq_2	GTGGTGCCTGTTGATTAAATTG
PPV1_Cap_seq_3	AACCAGAGGTAAGAAGATCGCC
PPV2_Cap_F	AGGTAAGCGGCCATGAGCG
PPV2_Cap_R	ACCTCTTTATACACGATGCGC
PPV2_Cap_seq_1	TCTACCGAAGTGGGAGGATCAG
PPV2_Cap_seq_2	CATGAGAGAGTGCCGATTATTG
PPV3_Cap_F	GGTAAGAAATCATGACAGCCG
PPV3_Cap_R	GTTAGCATTACAATTTGCGGGA
PPV3_Cap_seq_1	CTGGTAATCCTTTGGATAATGCTC
PPV3_Cap_seq_2	TTGGTGGAGATTTACCCTCCTC
PPV4_Cap_F	TGACGAAGCAGCTCTTCGAC
PPV4_Cap_R	ATCATCTGCGGTGTCTGGGT
PPV4_Cap_seq_1	GAAGATGTGTTCTCTCAGCG
PPV4_Cap_seq_2	ATTCTGTTGTGTACCCATAAGC
PPV5_Cap_F	GAAGTGGAGGAACAATGAGCT
PPV5_Cap_R	ATTATCTTCTCGCTCTAACACG
PPV5_Cap_seq_1	AGGACATCGCTACACAGGTC
PPV5_Cap_seq_2	ACCACGACAGGTGTATCATTAAAG
PPV6_Cap_F	GTACCCAGTTTCCTGACGAC
PPV6_Cap_R	ACTGTAGTAAGTGTATTGCTGG
PPV6_Cap_seq_1	TAAACAAGAAGGGCAGATGTCAAG
PPV6_Cap_seq_2	CACATGGCGGGTCTATCATGAC
PPV7_Cap_F	TCGGTGATCAATAAAGCGCCT
PPV7_Cap_R	ACTGGTTTAGCTTCTTATTTTCG

For phylogenetic analysis, capsid protein gene reference sequences from the NCBI GenBank database and the new sequences obtained in this study were aligned by Clustal Omega [36]. A maximum likelihood (ML) phylogenetic tree with a gamma distribution and invariant sites was constructed with MEGA X software [37] and the Tamura-Nei model using 1,000 bootstrap values.

### PCV2 and PRRSV detection in lung samples

Lung samples submitted to JBNU-VDC are routinely tested for PCV2 and PRRSV infection corresponding to client requests using the Prime-Q PCV2 PRRSV Detection Kit (GeNet Bio Inc., Daejun, Korea) according to the manufacturer’s instructions [32].

The PCV2 real-time PCR results for lung tissue samples were used to categorize individual pigs into PCV2-negative (ct > 35), LOW-PCV2-PIG (ct > 25 and ≤ 35) or HIGH-PCV2-PIG (ct ≤ 25) groups based on real-time PCR ct values. According to the previously established criteria for PCVAD based on the PCV2 ct value [23, 38], HIGH-PCV2-PIG samples were classified as PCVAD-suspected, and PCV2-negative and LOW-PCV2-PIG samples were combined and classified as non-PCVAD-suspected.

Based on the PRRSV real-time PCR results, lung tissue samples were considered to be PRRSV positive (ct ≤ 35) and PRRSV negative (ct > 35) and were categorized into the following groups: PRRSV-Neg, PRRSV1 single infection, PRRSV2 single infection, and PRRSV1&2 dual infection. For further investigation regarding the correlation between PPV and PRRSV infection, PRRSV1- and/or PRRSV2-infected pigs were all combined into the category PRRSV-Pos.

### Statistical analysis

For the data analysis, GraphPad Prism 7 (GraphPad, San Diego, CA, USA) was used for graph construction and statistical analysis. Differences in PPV1-PPV7 prevalence rates were determined by using the chi-square test by pairwise comparisons [23]. A two-tailed *p*-value of less than 0.05 was set as the statistically significant level.

## Results

### Prevalence of PPV1-PPV7 infections in Korea

In this study, 268 serum pool samples (pooled from 1192 serum samples) and 481 lung and 251 fecal samples were tested for PPV using multiplex PCR. PPV1, PPV2, PPV3, PPV4, PPV5, PPV6, and PPV7 were detected in all the sample types, except that PPV1 was not detected in serum samples (Table 3). Overall, when combining all the sample types, PPV2 was most prevalent in Korean swine herds (22.1%), followed by PPV6 (21.5%), PPV5 (14.3%), PPV7 (14.2%), PPV3 (11.6%), PPV4 (8.2%), and PPV1 (4.5%) (Table 3). By sample type, positive rates in lung samples were significantly higher than those in other sample types among PPV1, PPV2, PPV3, PPV5, PPV6, and PPV7, while that of PPV4 was similar among all the sample types (Table 3).

**Table 3:** Prevalence of porcine parvoviruses in Korean swine herds

Virus type	Number of positive/total sample (prevalence)			
	**serum**	**lung**	**feces**	**TOTAL**
PPV1	0/268^a*^ (0.0%)	38/481^b^ (7.9%)	7/251^c^ (2.8%)	45/1000 (4.5%)
PPV2	32/268^a^ (12.2%)	157/481^b^ (32.6%)	32/251^a^ (12.7%)	221/1000 (22.1%)
PPV3	11/268^a^ (4.2%)	84/481^b^ (17.5%)	21/251^a^ (8.4%)	116/1000 (11.6%)
PPV4	25/268 (9.5%)	41/481 (8.5%)	15/251 (6.0%)	82/1000 (8.2%)
PPV5	25/268^a^ (9.5%)	104/481^b^ (21.6%)	14/251^a^ (5.6%)	143/1000 (14.3%)
PPV6	51/268^a^ (19.4%)	133/481^b^ (27.7%)	31/251^c^ (12.4%)	215/1000 (21.5%)
PPV7	5/268^a^ (1.9%)	128/481^b^ (26.6%)	9/251^a^ (3.6%)	142/1000 (14.2%)

* Different superscripts (a, b, and c) within a row indicate a significantly (*p* < 0.05) different proportion of PPV-positive results among the different sample types

All PPV types were detected in suckling piglets, but the positive rates of all PPV types in the piglet group were significantly lower (< 5%) than those in the older age groups (Table 4). In weaners, PPV2 was the most commonly detected species (27.9%), followed by PPV6 (21.5%), PPV7 (18.6%), PPV5 (14.7%), PPV3 (11.2%), PPV4 (5.4%), and PPV1 (5.0%) (Table 4). In fatteners, PPV6 was the most common (36.5%), followed by PPV2 (26.1%), PPV5 (22.9%), PPV7 (17.7%), PPV3 (16.9%), and PPV1 (6.4%) (Table 4). In the sow or gilt group, the positive rates of PPV types were relatively (PPV1 and PPV3) or significantly (PPV2, PPV4, PPV5, PPV6, and PPV7) lower than those in the weaner and fattener groups (Table 4). PPV3 and PPV6 were the most common (10.5%) in the sow or gilt group, followed by PPV4 (7.0%), PPV2 (4.9%), PPV5 (3.5%), PPV1 (1.4%), and PPV7 (0.7%) (Table 4). Overall, the positive rates of all the PPV types were significantly higher among the weaner and fattener groups than among the suckling piglet group and/or the group of sows or gilts (Table 4 and Supplementary Fig. 1).

**Table 4:** Agewise prevalence of porcine parvoviruses in Korean swine herds

Virus type	Age group (weeks)			
	**Piglets (≤ 4)**	**Weaners (5–8)**	**Fatteners (≥ 9)**	**Sow/gilt**
PPV1	1/112^a*^ (0.9%)	26/516^bc^ (5.0%)	16/249^c^ (6.4%)	2/143^b^ (1.4%)
PPV2	5/112^a^ (4.5%)	144/516^b^ (27.9%)	65/249^b^ (26.1%)	7/143^a^ (4.9%)
PPV3	1/112^a^ (0.9%)	58/516^b^ (11.2%)	42/249^c^ (16.9%)	15/143^bc^ (10.5%)
PPV4	5/112^a^ (4.5%)	28/516^a^ (5.4%)	38/249^b^ (15.3%)	10/143^a^ (7.0%)
PPV5	4/112^a^ (3.6%)	76/516^b^ (14.7%)	57/249^c^ (22.9%)	5/143^a^ (3.5%)
PPV6	4/112^a^ (3.6%)	111/516^b^ (21.5%)	91/249^c^ (36.5%)	15/143^d^ (10.5%)
PPV7	1/112^a^ (0.9%)	96/516^b^ (18.6%)	44/249^b^ (17.7%)	1/143^a^ (0.7%)

*Different superscripts (a, b, c, and d) within a row indicate a significantly (*p* < 0.05) different proportion of PPV-positive results among different age groups

### Phylogenetic analysis of the capsid protein gene of Korean PPV1-PPV7

**Fig. 1:**
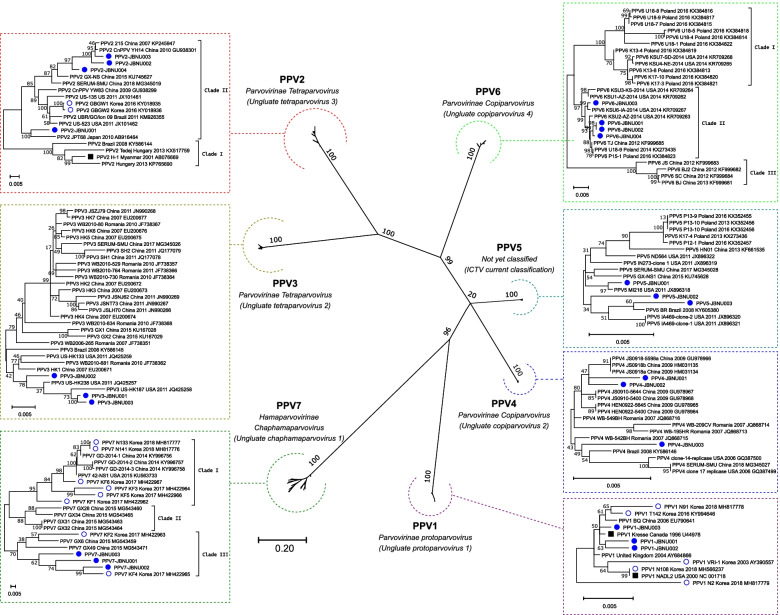
Maximum-likelihood tree analysis based on combined structural gene nucleotide sequences [VP2 (PPV1 and 3) and Cap (PPV2, PPV4, PPV5, PPV6, and PPV7)] constructed with the Tamura-Nei model, with a gamma distribution, invariant sites, and 1,000 bootstrap values, in the MEGA X program. Korean PPVs from this study and previous study are marked with blue filled circles and hollow circles, respectively

Based on the capsid protein gene of PPVs (VP2 gene for PPV1 and PPV3; capsid gene for PPV2, PPV4, PPV5, PPV6 and PPV7), seven well-defined groups were established that correspond to each PPV (Fig. 1). PPV2, PPV6 and PPV7 showed relatively higher genetic distances (> 5%) within the group than PPV1, PPV3, PPV4 and PPV5 (< 2%) (Table 5). For PPV1, PPV3, PPV4, PPV5, and PPV7, Korean PPV strains were genetically similar to globally identified strains. For PPV2, all the Korean strains were grouped into clade II, which is different from clade I, which is close to the H-1 strain found in Myanmar (Fig. 1). PPV6 and PPV7 could be divided into three distinct clades, and Korean PPV6 strains were grouped into clade II (Fig. 1). Korean PPV7 strains were grouped into clade I or clade III, but not clade II (Fig. 1).

**Table 5:** Genetic homology of the capsid protein gene of each PPV type and mean genetic distance between clades shown as percentages in nucleotides

	**Genetic homology (Mean ± S.D.) or genetic distance between clades (Mean ± S.D.) (%)**						
	**PPV1**	**PPV2**	**PPV3**	**PPV4**	**PPV5**	**PPV6**	**PPV7**
TOTAL	98.14—100 (99.29 ± 0.41)	92.24—99.87 (95.70 ± 1.93)	97.00—99.82 (98.28 ± 0.64)	98.89—100 (99.44 ± 0.28)	98.57—100 (99.25 ± 0.32)	94.53—100 (97.86 ± 1.31)	88.46—100 (92.52 ± 2.52)
within clade I	-	95.81—97.79 (96.68 ± 0.65)	-	-	-	97.32—99.97 (98.64 ± 0.81)	92.41—99.93 (95.66 ± 2.32)
within clade II	-	94.46—99.87 (96.92 ± 1.25)	-	-	-	99.07—100 (99.58 ± 0.23)	91.37—100 (95.23 ± 2.35)
within clade III	-	-	-	-	-	96.64—99.92 (98.26 ± 1.30)	90.19—96.41 (92.55 ± 1.47)
clade I vs. clade II	-	5.23—7.06 (6.53 ± 0.40)	-	-	-	1.30—3.38 (2.14 ± 0.54)	6.59—10.32 (8.71 ± 0.94)
clade I vs. clade III	-	-	-	-	-	3.36—5.47 (4.30 ± 0.57)	6.05—11.54 (9.08 ± 1.41)
clade II vs. clade III	-	-	-	-	-	2.89—3.51 (3.29 ± 0.16)	4.51—9.94 (8.44 ± 1.33)

### Association of PPV1-PPV7 with PCVAD and PRRSV

**Fig. 2:**
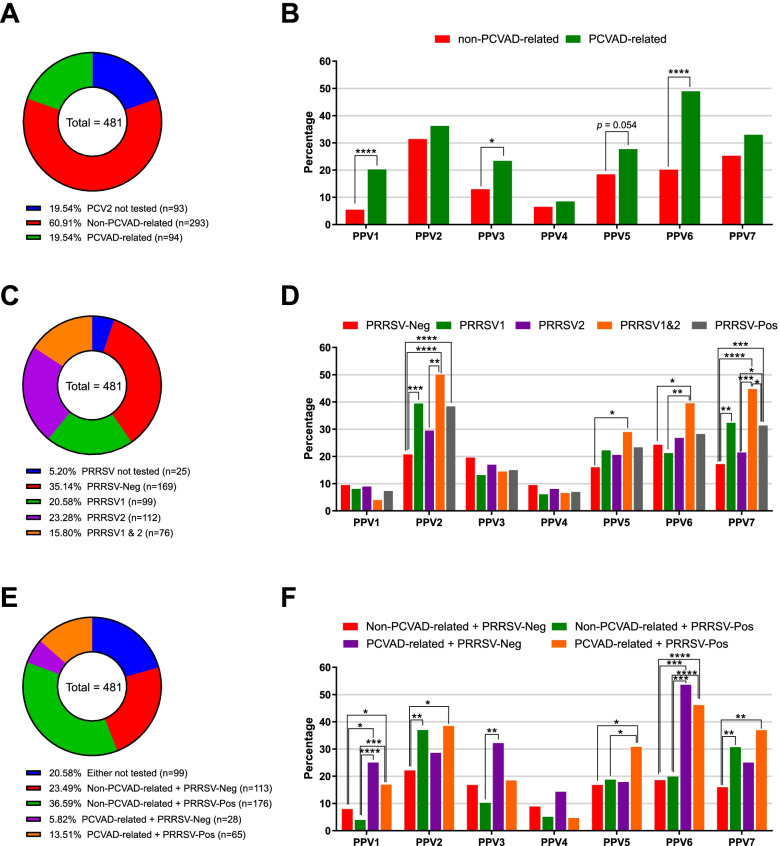
Positive rates of each PPV type associated with PCVAD and/or PRRSV infection. Lung samples were categorized and plotted into pie charts of (**a**) PCVAD status, (**c**) PRRSV infection status, and (**e**) PCVAD and PRRSV combined. Subsequently, positive rates of lung samples for each PPV type were plotted into bar charts based on (**b**) PCVAD status, (**d**) PRRSV infection status, and (**f**) PCVAD and PRRSV combined. Asterisks indicate significant differences (*p < 0.05; **p < 0.01, ***p < 0.001, ****p < 0.0001) between each status

Data regarding the detection of PCV2 and PRRSV in lung samples (*n* = 481) were gathered from the JBNU-VDC database. For PCV2, after excluding 93 lung samples (19.54%) due to no testing or lack of data, 293 lung samples (60.91%) were classified as non-PCVAD-suspected (ct > 25), and 94 as PCVAD-suspected (ct ≤ 25) (Fig. 2A). When the non-PCVAD-suspected and PCVAD-suspected samples were compared, the prevalence of PPV1 (*p* < 0.0001), PPV3 (*p* = 0.015) or PPV6 (*p* < 0.0001) was higher in the PCVAD-suspected samples than in the non-PCVAD-suspected samples (Fig. 2B). There were no significant differences for PPV2, PPV4, PPV5 or PPV7.

For PRRSV, 25 lung samples were excluded due to a lack of data (5.20%), and the rest of the samples were classified into the following categories: PRRSV-negative (35.14%, 169/481), PRRSV1-infected (20.58%, 99/481), PRRSV2-infected (23.28%, 112/481), and PRRSV1&2 coinfected (15.80%, 76/481) (Fig. 2C). A total of 287 lung samples were PRRSV1- and/or PRRSV2-positive (59.67%). When all the categories were compared, the prevalence of PPV2 (*p* < 0.0001) or PPV7 (*p* = 0.0009) was higher in PRRSV-positive samples than in PRRSV-negative samples (Fig. 2D). The prevalence of PPV5 or PPV6 was only higher in PRRSV1&2 coinfected samples than in PRRSV-negative samples (*p* = 0.0189 and *p* = 0.0152, respectively). There were no significant differences for PPV1, PPV3 or PPV4.

For systematic analysis of both PCV2 and PRRSV with PPV infection status, PCV2 and PRRSV detection data were combined. After excluding 99 lung samples (20.58%) that lacked PCV2 and/or PRRSV detection data, the lung samples were categorized into the following groups: non-PCVAD-suspected + PRRSV-neg (23.49%, 113/481), non-PCVAD-suspected + PRRSV-pos (36.59%, 176/481), PCVAD-suspected + PRRSV-neg (5.82%, 28/481), and PCVAD-suspected + PRRSV-pos (13.51%, 65/481) (Fig. 2E). When all the categories were compared, PPV1 and PPV6 were confirmed to be significantly more prevalent in the PCVAD groups regardless of their PRRSV infection status (Fig. 2F). The prevalence of PPV2 and PPV7 was significantly higher in PRRSV-positive samples regardless of PCVAD than in both non-PCVAD-suspected and PRRSV-negative samples. PPV5 was only significantly more prevalent in the group of both PCVAD-suspected and PRRSV-positive samples than in the group of both non-PCVAD-suspected and PRRSV-negative samples. The results were compared with previous studies [23–25, 29] and are summarized in Table 6.

**Table 6:** Summary of associations among PPV, PCVAD, and PRRSV infection together with earlier reports

	**This study (Korea)**	**Study from USA** **(Opriessnig et al., 2014)**	**Study from China** **(Qin et al., 2018)**	**Study from Poland** **(Milek et al., 2020)**	**Study from Mexico** **(Garcia‐Camacho et al., 2020)**
Study design	PPV, PCV2, and PRRSV detection in lungs	PPV and PCV2 detection in lungs	Virome analysis of PRDC-affected and healthy piglets	PPV and PCV2 detection in serum	PPV and PCV2 detection in lungs
Correlation with PPV1	Significantly more detected in PCVAD cases	Significantly more detected in PCVAD cases (*p* = 0.0048)	n/a	Significantly more detected in PCVAD cases	Not significant
Correlation with PPV2	Significantly more detected in PRRSV cases	Significantly more detected in PCVAD cases (*p* = 0.0002)	Significantly more detected in PRDC cases (*p* = 0.005)	Not significant	Not significant
Correlation with PPV3	Not significant	Not significant	Significantly more detected in PRDC cases (*p* < 0.001)	Significantly more detected in PCVAD cases	Not significant
Correlation with PPV4	Not significant	Not significant	Not significant (*p* = 0.33)	Not significant	Not significant
Correlation with PPV5	Significantly more detected in both PCVAD- & PRRSV-positive cases	Not significant	Not significant (*p* = 0.53)	Significantly more detected in PCVAD cases	Significantly more detected in PCVAD cases (p < 0.01)
Correlation with PPV6	Significantly more detected in PCVAD cases	n/a	Significantly more detected in PRDC cases (*p* < 0.001)	Significantly more detected in PCVAD cases	Significantly more detected in PCVAD cases (p < 0.01)
Correlation with PPV7	Significantly more detected in PRRSV cases	n/a	n/a	Not significant	n/a
Note		No PRRSV data	PCV2 and PRRSV were significantly more detected from PRDC-affected piglets	No PRRSV data Lower PCV2 ct value detected from PPV1- (p = 0.001) or PPV7- (p = 0.006) positive samples	No PRRSV data

*n/a,* Not applicable; *PRDC,* Porcine respiratory disease complex

## Discussion

The role of novel PPVs in pig health is difficult to understand. In contrast to those for PPV1, challenge experiments with novel PPVs (PPV2-PPV7) cannot be performed, as these viruses, except for PPV2 [39], have never been cultured in vitro [10]. Thus, the significance of the viruses can only be speculated from their detection at the DNA level. Therefore, our study performed PPV detection from serum, lung, and fecal samples from groups of pigs at different ages collected nationwide. Previous studies have attempted to find a link between PPV infection and PCVAD, we analyzed PCV2 and PRRSV detection data, which were obtained by real-time PCR, together with PPV prevalence to investigate the connection between novel PPVs and other common pathogens (PCV2 and PRRSV) that are related to PRDC. However, the diagnostic laboratory samples used in this study might have be subjected to predisposed pre-screening non-probability bias and may not be representative of a random pool of pigs on farms.

Among all 1000 samples investigated regardless of sample type, PPV2 (22.1%) and PPV6 (21.5%) were detected at relatively high frequencies, followed by PPV5 (14.3%), PPV7 (14.2%), PPV3 (11.6%), PPV4 (8.2%) and PPV1 (4.5%) (Table 3). Among the sample types, lung samples showed significantly higher detection rates of PPVs than serum or fecal samples, except for PPV4 (Table 3). This result may reflect the generally known tropism of parvoviruses for mitotically active tissues or indicate that differences in capsid protein genes may differentiate the host tissue tropism of different PPVs, as reported for other parvoviruses (Fig. 1 and Table 5) [18]. Although different detection rates of PPVs in different studies may have been influenced by various sample types and testing protocols, the positive rates of PPVs in this study were similar to or slightly different from those of previous reports from other countries (Supplementary Table 1) [7, 8, 10–12, 15–17, 19, 23, 30], indicating that novel PPVs are widespread in Korean pig herds.

The patterns of overall low prevalence in suckling piglets and adult female pigs and significantly high prevalence in weaners and fatteners were observed for all PPV types (Table 4). For PPV1, the pattern can be explained by the common vaccination protocol for sows and the passive immunity transferred to their progeny followed by loss of maternal antibodies, which leads to significantly increased detection in fattening periods [10, 40]. Similarly, the same patterns observed in novel PPVs (PPV2-PPV7) were consistent with those identified in earlier investigations [10, 15–17] and can be assumed to indicate that passive immunity against those PPVs is protective for piglets and that infections with those PPVs persist until the late fattening period, generating a possible circulation cycle at the farm level [10, 15, 17].

PCV2 is a small DNA virus that is considered an important pig virus that has caused substantial economic loss if uncontrolled [21]. As parvoviruses exhibit tropism toward mitotically active tissues, it has been speculated that PPVs enhance PCV2 replication by stimulating host cell division and enzyme production [23]. Based on reports suggesting that PPV1 enhances the severity of PCVAD [22], several studies have been performed to infer the associations between PPV2-PPV7 and PCVAD. Opriessnig et al. [23] and Novosel et al. [20] suggested an association between PPV2 and PCV2, Li et al. [28] and Miłek et al. [24] between PPV3 and PCV2, Cibulski et al. [26] between PPV4 and PCV2, Garcia-Camacho et al. [25] and Miłek et al. [24] between PPV5 and PPV6 and PCV2, and Xing et al. [14] and Wang et al. [27] between PPV7 and PCV2. In this study, when only PCV2 was considered, PPV1, PPV3 and PPV6 showed significantly higher prevalence rates in lung homogenates with suspected PCVAD positivity (Fig. 2A and 2B).

However, the aforementioned reports considered only PCV2 coinfection associations and not PRRSV. PRRSV is a small RNA virus that is highly prevalent in the global swine industry, posing a great economic threat by causing respiratory disease in pigs often together with PCV2 in the form of PRDC [21, 33]. In a recent metagenomics analysis of lung samples from PRDC-affected piglets and healthy piglets, PPV2, PPV3, and PPV6 were significantly associated with PRDC, and PCV2 and PRRSV were also significantly more frequently detected [29]. Another viral metagenomics study revealed that PPV6 was found in 13% of pigs with PRRSV viremia [30]. In the current study, when considering only PRRSV, clear coinfection associations of PPV2 and PPV7 with PRRSV infection were evident in lung homogenates (Fig. 2C and 2D). The prevalence of PPV2 and PPV7 was significantly higher in PRRSV tissues regardless of PRRSV1 and/or PRRSV2. PPV5 and PPV6 were significantly higher only in the case of coinfection of PRRSV1 and PRRSV2.

Therefore, it is quite obvious that both PCV2 and PRRSV should be considered together when determining coinfection associations with PPVs, at least in the case of lung samples, where PRDC occurs. In this study, when considering both pathogens, PPV1 and PPV6 showed a clear coinfection association with PCVAD regardless of PRRSV infection, and PPV2 and PPV7 showed a clear coinfection association with PRRSV regardless of PCVAD (Fig. 2E and 2F). PPV5 was significantly detected in both PCVAD-suspected and PRRSV-infected samples. When compared with previous studies that systematically analyzed the coinfection association of PPVs and PCVAD from archived tissue samples confirmed as positive for PCVAD or PRDC [23, 25, 29] and from serum samples with subclinical infection of PCV2 [24], PPV1 and PPV6 were consistently correlated with PCVAD, as identified in the current study (Table 6). However, PPV2 and PPV7 were identified to be positively correlated with PRRSV rather than PCV2 in the current study, which is different from other reports. In fact, PPV2 is a relatively well-studied virus compared to other novel PPVs (PPV3-PPV7). The coinfection association of PPV2 with PCV2 was speculated by several studies [4, 19, 23], and a recent study revealed that PPV2 localization was observed in lung lymphocytes from postweaning multisystemic wasting syndrome (PMWS)-affected lungs, especially in immature B lymphocytes and/or NK lymphocytes, without the participation of other respiratory pathogens using in situ polymerase chain reaction (PCR)/IS-PCR, implying a potential participation of PPV2 in clinical disease [20]. However, most importantly, experimental coinfection of PCV2 and PPV2 showed little or no effect on PCV2 challenge virulence [39]. It could be expected that previous evidence indicating a coinfection association of PPV2 with PCVAD might have failed to provide an unambiguous picture due to the absence of a PRRSV infection background. Although other commonly found pathogens in PRDC, such as swine influenza virus (SIV) or torque teno sus virus (TTSuV), could not be considered in this study [29], our results could elucidate the adverse effects of novel PPVs (PPV2-PPV7) on the course of PRDC, in which PCV2 and PRRSV are mainly involved. Further research on experimental single- and dual-infection PPVs, PCV, and PRRSV is needed to investigate the role and pathogenesis of PPVs in PRDC.

## Conclusion

To date, this is one of the largest studies on the prevalence of novel PPVs and the first study to systematically investigate the coinfection association not only between PPVs and PCV2 but also PRRSV. Our results confirm that novel PPVs are common in Korean pig herds. Among all the sample types and age groups investigated, PPV detection was highest in lung samples and fatteners. Detection patterns of PPVs suggest passive immunity and chronic characteristics of the infection, as these viruses were detected significantly less frequently in piglets and adult female pigs and persisted until a late period of fattening. Regarding PRDC, coinfection associations of PPV1 and PPV6 with suspected PCVAD (regardless of PRRSV) and that of PPV2 and PPV7 with PRRSV infection (regardless of suspected PCVAD) were identified. PPV5 was significantly more frequently detected in high-risk of PRDC cases where PCV2 and PRRSV were both infected. This report updates the knowledge regarding novel PPVs and highlights the need for further research on the pathogenesis of PPVs in PRDC through experimental challenge. 

## Supplementary Information

**Additional file 1: Supplementary Figure 1.** Percentage of porcine parvoviruses 1-7 (PPV1-PPV7) positive samples from piglets, weaners, fatteners and sow or gilts in different diagnostic materials (serum, lungs and feces).

**Additional file 2: Supplementary Table 1.** Comparison of detection rates of porcine parvoviruses, in different age groups of pigs and different materials, between this study and earlier reports. “-” indicate negative results (this study) or lacking data (earlier study).

## Data Availability

All data generated and analyzed in this study are included in this article and supplemental materials. The nucleotide sequence data that support the findings of this study are openly available in the GenBank database at http://www.ncbi.nlm.nih.gov/genbank/, accession numbers MZ491178-MZ491200.

## Abbreviations

PPV: Porcine Parvovirus
PCV2: Porcine Circovirus 2
PRRSV: Porcine Reproductive and Respiratory Syndrome Virus
PRDC: Porcine Respiratory Disease Complex
PCVAD: PCV2-Associated Diseases
SIV: Swine Influenza Virus
JBNU-VDC: Jeonbuk National University Veterinary Diagnostic Center
ML: Maximum Likelihood
Ct: Cycle Threshold
TTSuV: Torque Teno Sus Virus
