# Supplementary material for: Prevalence of porcine parvovirus 1 through 7 (PPV1-PPV7) and co-factor association with PCV2 and PRRSV in Korea
Source: BMC Vet Res. 2022 Apr 9;18:133. doi: 10.1186/s12917-022-03236-1 (PMC8994367; doi:10.1186/s12917-022-03236-1)
Supplement: Supplementary file 1 — Additional file 1: Supplementary Figure 1. Percentage of porcine parvoviruses 1-7 (PPV1-PPV7) positive samples from piglets, weaners, fatteners and sow or gilts in different diagnostic materials (serum, lungs and feces). [file 12917_2022_3236_MOESM1_ESM.pdf]

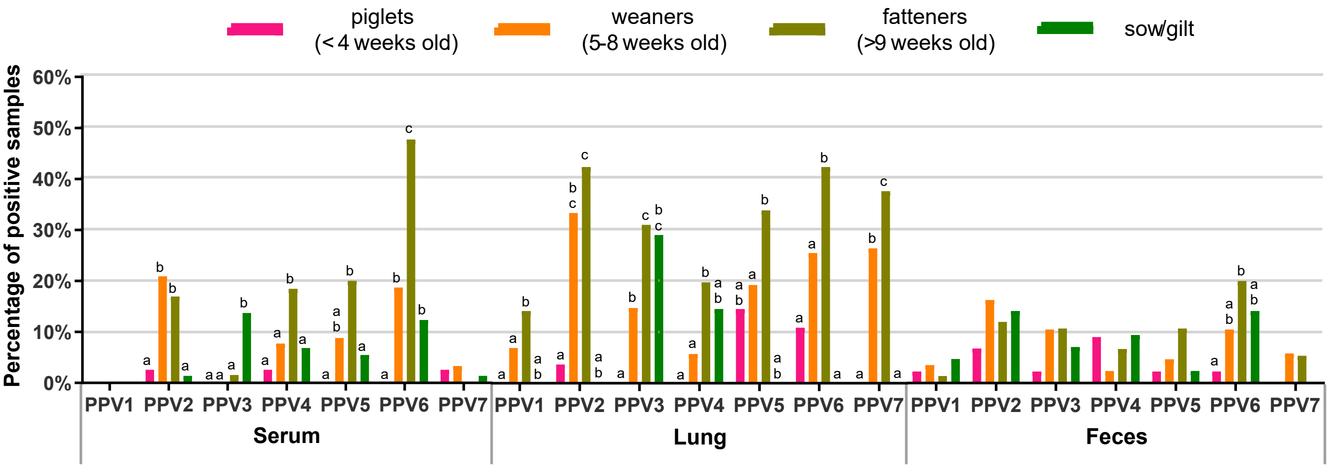

Additional file 1.pdf: Supplementary Figure 1. Percentage of porcine parvovirus 1-7 (PPV1-PPV7)-positive samples from piglets (n = 112), weaners (n = 516), fatteners (n = 249), and sows/gilts (n = 123) in different diagnostic materials (serum, lung, and feces). Statistically significant differences ($p < 0.05$, chi-square test) between age groups in diagnostic materials, within bars grouped for each PPV type, are marked with superscripts on the top of the bars in the chart (a, b, and c)
